# Doctors of Pharmacy

**Published:** 1876-05

**Authors:** 


*Doctors of Pharmacy.*—In reply to our correspondent, Dr. C—, we thank him for the information in regard to the proposal of the Tennessee College of Pharmacy to examine and graduate an applicant without his attending the lectures, but we do not care now to discuss this matter. Possibly, the college may have a legal right under their charter to this irregular procedure. It is a matter not so much for the consideration of the journalist as for the action of the American Pharmaceutical Association, which, jealous of its rights and of its honor, will doubtless take cognizance of all such irregularities, whether based upon legal enactment or upon individual authority.

For want of space and proper information we cannot at present answer his inquiries in regard to the attachment of a Pharmaceutical and a Dental Chair to the Atlanta Medical College. We will endeavor to procure the necessary information for our next issue; after which our correspondent can have space in our journal for the statement of his views on this matter; provided, however, that personalties be avoided, and the question be discussed alone upon the principles involved.

